# Cytostatic and anti-angiogenic effects of temsirolimus in refractory mantle cell lymphoma

**DOI:** 10.1186/1756-8722-3-30

**Published:** 2010-09-09

**Authors:** Li Wang, Wen-Yu Shi, Zhi-Yuan Wu, Mariana Varna, Ai-Hua Wang, Li Zhou, Li Chen, Zhi-Xiang Shen, He Lu, Wei-Li Zhao, Anne Janin

**Affiliations:** 1 Shanghai Institute of Hematology, Shanghai Rui Jin Hospital, Shanghai Jiao Tong University School of Medicine, Shanghai, China; 2 Inserm, U728, Pôle de Recherches Franco-Chinois, Paris, France; 3 Department of Radiology, Shanghai Rui Jin Hospital, Shanghai Jiao Tong University School of Medicine, Shanghai, China; 4 University Paris Diderot, Paris, France

## Abstract

Mantle cell lymphoma (MCL) is a rare and aggressive type of B-cell non-Hodgkin's lymphoma. Patients become progressively refractory to conventional chemotherapy, and their prognosis is poor. However, a 38% remission rate has been recently reported in refractory MCL treated with temsirolimus, a mTOR inhibitor.

Here we had the opportunity to study a case of refractory MCL who had tumor regression two months after temsirolimus treatment, and a progression-free survival of 10 months. In this case, lymph node biopsies were performed before and six months after temsirolimus therapy. Comparison of the two biopsies showed that temsirolimus inhibited tumor cell proliferation through cell cycle arrest, but did not induce any change in the number of apoptotic tumor cells. Apart from this cytostatic effect, temsirolimus had an antiangiogenic effect with decrease of tumor microvessel density and of VEGF expression. Moreover, numerous patchy, well-limited fibrotic areas, compatible with post-necrotic tissue repair, were found after 6-month temsirolimus therapy. Thus, temsirolimus reduced tumor burden through associated cytostatic and anti-angiogenic effects.

This dual effect of temsirolimus on tumor tissue could contribute to its recently reported efficiency in refractory MCL resistant to conventional chemotherapy.

## Background

Mantle cell lymphoma (MCL) is an aggressive B-cell non-Hodgkin's lymphoma (NHL), representing about 6% of NHL cases. T(11;14)(q13;q32) chromosomal translocation, one of the most important cytogenetic abnormalities of MCL, juxtaposes genes of cyclin D1 and of immunoglobulin heavy chain, inducing cyclin D1 over-expression and cell cycle deregulation [1]. Thus, cyclin D1 over-expression and/or the t(11;14)(q13;q32) translocation are hallmarks of MCL, included in current WHO guidelines for MCL diagnosis [2]. MCL patients are usually diagnosed at an advanced stage (III or IV). They become progressively refractory to conventional chemotherapy, and have a poor overall survival [3]. Therefore, alternative therapeutic strategies are actively studied.

The mammalian Target Of Rapamycin (mTOR) is a serine/threonine protein kinase. It plays an important role in cell growth, protein synthesis, and cell-cycle progression [4]. Since mTOR pathway is constitutively activated in MCL, it could be a potent therapeutic target for this disease [5]. Recent clinical trials showed that temsirolimus (Wyeth Pharmaceutical, Philadelphia, PA), a mTOR inhibitor, induced a 38% response rate and a prolonged progression-free survival (PFS) of 3.4-6.9 months in refractory MCL patients [6,7]. We studied here a refractory MCL patient, who had tumor regression under temsirolimus treatment.

## Case Presentation

**Figure 1:**
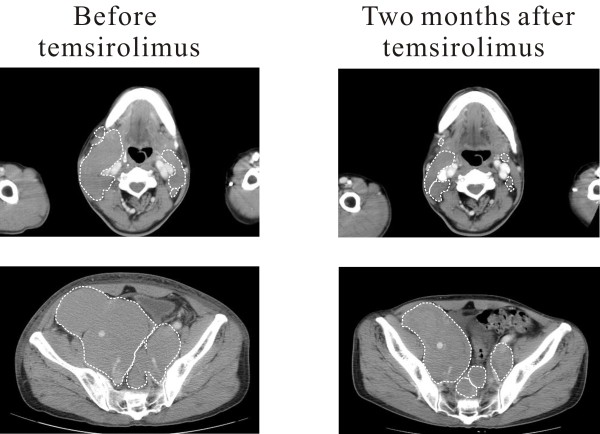
**Computed tomography images of MCL**. Areas of major lesions (surrounded by white broken lines) significantly regressed after two months of temsirolimus treatment.

A 53-year-old male with generalized lymphadenopathy and fatigue, was diagnosed as MCL on inguinal lymph node biopsy. After 10 cycles of CHOP and 2 cycles of E-CHOP, lymph nodes bulged. Disease was still progressing after 2 cycles of R-ICE. Therefore, R-ICE was stopped. The patient was recruited in phase III study of temsirolimus (number: 3066K1-305-WW) on August 2006 but was randomized in investigator's choice group. According to the protocol, fludarabine 25 mg/m^2 ^was infused daily for 5 days, and it was repeated every 28 days. After 8 cycles, fludarabine had to be stopped because of severe bone marrow inhibition on March 2007. One year later, enlarged iliac lymph-node compressed ureter, causing renal dysfunction with elevated blood creatinine. To confirm the diagnosis of recurrence, a biopsy of enlarged right cervical lymph node was performed and the place was noted on CT scan. After confirmation of the MCL recurrence, the patient was permitted to enter the temsirolimus treatment group on March 2008. He received temsirolimus 175 mg/week for 3 weeks, followed by weekly doses of 75 mg. Circulation blood count was monitored weekly, CT scan and serum chemistry every other month. Temsirolimus was suspended, when absolute neutrophil count <1000/μl, or hemoglobin <8 g/dl, or platelet <50000/μl. According to the response criteria for non-Hodgkin's lymphoma[8] we use in our hospital, six of the largest dominant nodes or nodal masses were measured. The sum of dimensions of these six nodal masses was recorded before temsirolimus as well as every other month under temsirolimus treatment. Other lesions were recorded but not measured. After 2 months of temsirolimus treatment, a 33% regression of the sum of dimensions was observed by CT scan (Figure 1). Meanwhile, renal function recovered and blood creatinine returned to normal level. However, lymph nodes enlargement was still present on CT scan after 6 months of temsirolimus. To assess the extent of the therapeutic effect, and to detect a possible early recurrence, a second biopsy of the same right cervical lymph node was performed but in a different direction. Informed consent was provided according to the Declaration of Helsinki. Disease remained stable until January 2009 when CT scan showed a cervical lymph node behind the right jugular vein bulged. Temsirolimus was then stopped. No further biopsy was taken. Patient then received arsenic combined with thalidomide and chlorambucil treatment. On March 2009, all lymph nodes enlarged, and disease still progressed after 3 cycles of bortezomib. The patient finally died of severe bone marrow inhibition and pulmonary infection after hyperCVAD treatment on October 2009.

During temsirolimus treatment, leukopenia and thrombocytopenia occasionally occurred, and disappeared after one week of treatment suspension. No sign of thrombosis was observed.

Cyclin D1, the hallmark of MCL, is the down stream target of mTOR. Its expression was assessed by immunohistochemical staining (Dako; Glostrup, Denmark; dilution 1:100) on the two successive biopsies. Tumor cell proliferation was assessed by Ki67 (Dako; dilution 1:100), apoptosis by cleaved caspase-3 (Cell signaling; MA, USA; dilution 1:50), microvessel density (MVD) by CD31 (Dako; dilution 1:50), and angiogenesis cytokine expression by VEGF-A (R&D system; MN, USA; dilution 1:200). Irrelevant isotypic antibodies and absence of primary antibodies were used as controls. Immunostained cells were counted on 5 different microscopic fields at ×400 magnification, out of fibrotic and necrotic areas, the count including a minimum of 1000 cells. Fibrotic areas were randomized photographed at ×200 magnification for five fields and analysed with Cell Software (Olympus, Tokyo). The ratio between fibrotic areas and tumor areas gave the relative fibrotic area. Differences between analyses before and after temsirolimus were assessed with Wilcoxon signed-rank test. Two-sided *P *< 0.05 was considered to be significant.

**Figure 2:**
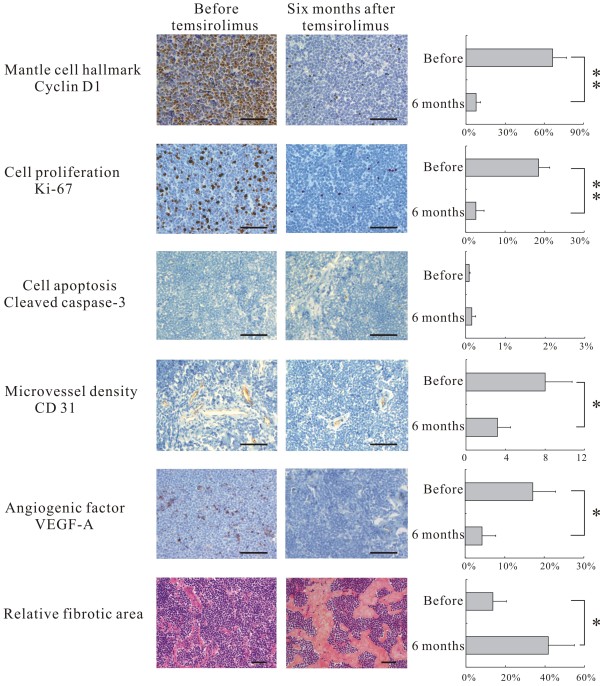
**Immunohistostainings and histological analysis of the lymph node biopsies before and six months after temsirolimus**. Quantitative studies showed a significant decrease of cyclin D1, cell proliferation, microvessel density and VEGF-A expression as well as a significant increase in fibrosis after six months of temsirolimus. Cleaved caspase-3 positive cell counts remained unchanged. Bar, 50 μm. * P < 0.05, ** P < 0.01

Comparison between the 2 biopsies, before and after temsirolimus, showed a significant decrease of cyclin D1 (*P *< 0.01), and Ki67 (*P *< 0.01). But there was no change in apoptotic cell counts (*P *= 0.15). VEGF-A expression (*P <*0.05) and microvessel density (*P *< 0.05) were also significantly decreased after temsirolimus therapy. Numerous patchy, well-limited fibrotic areas were observed within the tumor. Relative fibrotic area significantly increased after temsirolimus (*P *< 0.05) (Figure 2).

## Discussion and conclusion

The use of m-TOR inhibitor in MCL is an emerging therapy [7], but its in vivo anti-tumor mechanism is not yet fully explained. In this refractory MCL case, temsirolimus was able to induce tumor regression as well as a progression-free survival of 10 months. Tissue analyses before and after temsirolimus showed the direct cytostatic effect of this mTOR inhibitor through cell cycle arrest, as demonstrated by down-regulation of cyclin D1 and Ki67 in lymphoma cells, and the absence of apoptotic change. This cytostatic effect observed on human biopsies is in agreement with experimental results reported in temsirolimus-treated breast and acute leukemia cell lines [9,10]. However, temsirolimus significantly reduced tumor burden in our refractory MCL case, an effect difficult to link only to its cytostatic properties. Further assessment of its efficiency on lymphoma tissue showed that the tumor microvessel density and the VEGF-A expression were both significantly reduced after treatment. On the same biopsies, we also found patchy, well-limited fibrotic areas, compatible with post-necrotic tissue repair [11]. Along this line, tumor infarct and necrosis linked to tumor microvessel thrombi have been reported in xenografted pancreas and colon cancer treated by mTOR inhibitor [12]. Reduction of microvessel density and of VEGF-A expression were also found in another series of xenografted breast cancers [10]. Temsirolimus could thus reduce tumor burden through a direct cytostatic effect on the tumor cells, but also through an associated effect on tumor angiogenesis.

This dual effect of temsirolimus on tumor tissue could contribute to its recently reported efficiency in refractory MCL resistant to conventional cytotoxic drugs. On the long term, this supports the evaluation of anti-angiogenic drugs in refractory MCL.

## Abbreviations

MCL: mantle cell lymphoma; PFS: progression-free survival; CHOP: Cyclophosphamide, Hydroxydaunorubicin, Vincristine, and Prednisone; R-CHOP: Rituximab associated with CHOP; ICE: Ifosfamide, Carboplatin, and Etoposide; E-CHOP: Etoposide associated with CHOP; R-ICE: Rituximab associated with ICE; hyperCVAD: cyclophosphamide, vincristine, adriamycin, dexamethasone; CT: computed tomography

## Consent

Written informed consent was obtained from the patient for publication of this case report and any accompanying images. A copy of the written consent is available for review by the Editor-in-Chief of this journal.

## Competing interests

The authors declare that they have no competing interests.

## Authors' contributions

LW and WYS collected clinical data, performed statistical analysis and contributed equally to this work. ZYW and AHW performed radiological analysis. MV and AJ performed pathological analysis. LZ, LC, ZXS collected clinical data. HL, WLZ and AJ wrote the manuscript. All authors read and approved the final manuscript.
